# Clinical, imaging, and molecular analysis of pediatric pontine tumors lacking characteristic imaging features of DIPG

**DOI:** 10.1186/s40478-020-00930-9

**Published:** 2020-04-23

**Authors:** Jason Chiang, Alexander K. Diaz, Lydia Makepeace, Xiaoyu Li, Yuanyuan Han, Yimei Li, Paul Klimo, Frederick A. Boop, Suzanne J. Baker, Amar Gajjar, Thomas E. Merchant, David W. Ellison, Alberto Broniscer, Zoltan Patay, Christopher L. Tinkle

**Affiliations:** 1 Department of Pathology, St. Jude Children’s Research Hospital, Memphis, TN USA; 2 Department of Radiation Oncology, St. Jude Children’s Research Hospital, Memphis, TN USA; 3 Department of Radiation Oncology, Duke University Medical Center, Durham, North Carolina USA; 4 University of Tennessee Health Science Center, Memphis, TN USA; 5 Department of Biostatistics, St. Jude Children’s Research Hospital, Memphis, TN USA; 6 Department of Surgery, St. Jude Children’s Research Hospital, Memphis, TN USA; 7 Department of Neurosurgery, University of Tennessee Health Science Center, Memphis, TN USA; 8 Department of Developmental Neurobiology, St. Jude Children’s Research Hospital, Memphis, TN USA; 9 Department of Oncology, St. Jude Children’s Research Hospital, Memphis, TN USA; 10 Department of Pediatrics, University of Pittsburgh Medical Center, Pittsburgh, PA USA; 11 Department of Diagnostic Imaging, St. Jude Children’s Research Hospital, Memphis, TN USA

**Keywords:** Atypical DIPG, Biopsy, Histopathology, Univariable/multivariable analysis, H3 K27M

## Abstract

Diffuse intrinsic pontine glioma (DIPG) is most commonly diagnosed based on imaging criteria, with biopsy often reserved for pontine tumors with imaging features not typical for DIPG (atypical DIPG, ‘aDIPG’). The histopathologic and molecular spectra of the clinical entity aDIPG remain to be studied systematically. In this study, thirty-three patients with newly diagnosed pontine-centered tumors with imaging inconsistent with DIPG for whom a pathologic diagnosis was subsequently obtained were included. Neoplasms were characterized by routine histology, immunohistochemistry, interphase fluorescence in situ hybridization, Sanger and next-generation DNA/RNA sequencing, and genome-wide DNA methylome profiling. Clinicopathologic features and survival outcomes were analyzed and compared to those of a contemporary cohort with imaging features consistent with DIPG (typical DIPG, ‘tDIPG’). Blinded retrospective neuroimaging review assessed the consistency of the initial imaging-based diagnosis and correlation with histopathology. WHO grade II-IV infiltrating gliomas were observed in 54.6% of the cases; the remaining were low-grade gliomas/glioneuronal tumors or CNS embryonal tumors. Histone H3 K27M mutation, identified in 36% of the cases, was the major prognostic determinant. H3 K27M–mutant aDIPG and H3 K27M–mutant tDIPG had similar methylome profiles but clustered separately from diffuse midline gliomas of the diencephalon and spinal cord. In the aDIPG cohort, clinicoradiographic features did not differ by H3 status, yet significant differences in clinical and imaging features were observed between aDIPG without H3 K27M mutation and tDIPG. Neuroimaging review revealed discordance between the classification of aDIPG and tDIPG and did not correlate with the histology of glial/glioneuronal tumors or tumor grade. One patient (3.1%) developed persistent neurologic deficits after surgery; there were no surgery-related deaths. Our study demonstrates that surgical sampling of aDIPG is well-tolerated and provides significant diagnostic, therapeutic, and prognostic implications, and that neuroimaging alone is insufficient to distinguish aDIPG from tDIPG. H3 K27M-mutant aDIPG is epigenetically and clinically similar to H3 K27M-mutant tDIPG.

## Introduction

Despite the re-emergence of diagnostic biopsy at several centers over the past decade, diffuse intrinsic pontine glioma (DIPG) remains largely a clinical diagnosis based on characteristic features on conventional MRI [1, 2]. Although the imaging criteria used to define a classical or 'typical' DIPG (tDIPG) vary to some extent, and there are inconsistencies in the interpretation of the images [3], the general consensus radiographic features of tDIPG include a T1-hypointense and T2-hyperintense tumor involving at least 50% of the pons by cross-sectional area [4–6]. It has been argued that MR imaging interpreted using these criteria provides sufficient information to establish a diagnosis of tDIPG reliably, obviating the risk associated with tissue sampling [4, 7]. Clinical characteristics in the form of stereotypic acute neurologic symptoms are sometimes used with imaging to define tDIPG [5, 6, 8], yet these characteristics too are variable and are rarely used as eligibility criteria in modern clinical trials. In many centers in the United States, biopsy is reserved for patients with a clinical diagnosis of 'atypical' DIPG (aDIPG), i.e., pontine tumors in which the above imaging features are absent or incomplete [9]. These patients have traditionally been considered separate from patients with tDIPG for therapy or research purposes [10].

Molecular profiling of tDIPG [11] has resulted in a newly defined pathologic entity, H3 K27M–mutant diffuse midline glioma (DMG), which represents approximately 80% of radiographically recognized tDIPG [12]. However, the clinical entity ‘aDIPG’ has not been systematically studied, the attendant risks of biopsy in these patients have not been formally evaluated, and the extent to which pontine DMG manifesting as aDIPG is biologically and clinically distinct from tDIPG is poorly defined [13]. We sought to address these issues through a comprehensive analysis of the clinical, MR imaging, histopathologic, and molecular features of 33 patients with a clinical diagnosis of aDIPG who subsequently underwent tumor tissue sampling and treatment at our institution. We compared the characteristics of these patients to those of a contemporary cohort of 100 patients with newly diagnosed tDIPG to identify variables that correlated with the clinical diagnosis of aDIPG. Finally, to evaluate the consistency of radiographic diagnosis of aDIPG and to correlate the diagnosis with final pathology, we conducted a blinded neuroradiology review.

## Materials and methods

### Patient cohort

Thirty-three treatment-naïve pediatric patients with a reported pontine-centered lesion with imaging features [14–16], with or without clinical presentation [5], atypical of tDIPG at initial diagnostic workup, and who underwent histologic tumor evaluation and were treated at St. Jude Children’s Research Hospital (St. Jude) between 2003 and 2018 were retrospectively identified. Two patients underwent diagnostic biopsy elsewhere following recommendations based on atypical imaging features. One patient underwent autopsy at St. Jude after deferring recommended diagnostic biopsy. The remaining 30 patients underwent a diagnostic biopsy or resection at St. Jude following the consensus recommendation after multidisciplinary review. Of the 29 patients who underwent biopsy, a total of 34 diagnostic surgical procedures were performed, including 26 needle biopsies using a transcerebellar approach and eight craniectomies. All surgical procedures used stealth frameless (31) or frame-based (6) stereotactic MRI-guidance.

A comparison cohort comprised 100 pediatric patients with tDIPG, based on a central review of diagnostic MRIs (Z.P. and C.L.T), who were treated at our institution between 2006 and 2014. Typical DIPG was defined radiographically as a poorly defined tumor with mass effect occupying ≥75% of the axial diameter of the pons that was hypointense on T1-weighted MR images and hyperintense on T2-weighted images. Patient demographics, treatment, and outcome data were extracted from medical records. Specific imaging features were assessed by trained observers (A.K.D. and L.M) under the supervision of a neuroradiologist (Z.P.) or radiation oncologist (C.L.T). This study was approved by our institutional review board (approval no. XPD18–008/XPD18–048/XPD19–0061).

### Histopathology review and molecular studies

Histopathology was centrally reviewed by a neuropathologist specializing in pediatric CNS tumors (J.C.). Standard hematoxylin and eosin histopathologic preparations from each case were supplemented by immunohistochemistry on 5-μm formalin-fixed, paraffin-embedded (FFPE) tissue sections. Monoclonal anti–histone H3 K27M antibody (RevMab Biosciences, #31–1175-00, clone RM192; diluted 1:250) was used to identify tumors expressing K27M-mutant histone H3. A monoclonal antibody (Cell Signaling Technology, # 9733, clone C36B11; diluted 1:200) was used to confirm the loss of trimethylation of the histone H3 K27 residue in K27M-mutant tumors. Sanger sequencing using variant-specific primers (Supplementary Table 1) was then used to identify the mutant histone H3 variant. Chromosome 7q34 duplication (a marker for *KIAA1549–BRAF* fusion), *MYB* rearrangement, and amplification of the microRNA cluster on chromosome 19q13.4 (C19MC) were detected by interphase fluorescence in situ hybridization (iFISH) with probes developed in-house (information available upon request). Whole-genome sequencing (WGS), whole-exome sequencing (WES), and RNA sequencing (RNA-seq) were performed using genomic DNA or total RNA extracted from snap-frozen or FFPE tissue. Sequencing results were analyzed using an institutionally established pipeline in a Clinical Laboratory Improvement Amendments (CLIA)–certified laboratory. Single-nucleotide variants were discovered using the Bambino variant-detection program, annotated and ranked by putative pathogenicity, and then manually reviewed.

### Genome-wide DNA methylation profiling and analysis

Analysis of genome-wide DNA methylation profiles was performed as previously described [17–20]. Reference methylation profiles of IDH-mutant astrocytomas and H3 K27M–mutant DMG of the diencephalon and spinal cord were downloaded from a publicly available database for comparison [21]. Raw signal intensities were normalized by performing background correction and a dye-bias correction for both color channels with the functional normalization method. The following filtering criteria were applied: removal of probes targeting the X and Y chromosomes; removal of probes containing single-nucleotide polymorphisms; and removal of probes not mapping uniquely to the human reference genome (hg19), allowing for one mismatch, after removal of poor-quality (*P* > 0.01) and failed probes. Beta values of the 5000 most variable CpG sites were derived for further analysis. T-distributed stochastic neighbor embedding (t-SNE) analysis was performed in R by using the Rtsne package v.0.13 with theta = 0.0. Agglomerative nesting hierarchical clustering analysis was performed using cluster package v.2.0.7–1 with Euclidean distances and a generalized average method.

### Blinded neuroimaging review

De-identified baseline anatomic MR images were retrospectively re-reviewed by a neuroradiologist specializing in pediatric brainstem tumors (Z.P.) who was blinded to the clinical presentation and histologic diagnosis. MRI studies included non-enhanced T1- and T2-weighted images, contrast-enhanced T1-weighted images, and diffusion-weighted images. Occasionally contrast-enhanced T2-FLAIR and T2 or susceptibility-weighted images were also available. Tumors were independently classified by radiographic patterns according to the following subjective classification schema: 1) *Typical DIPG* (an intra-axial expansile lesion centered on the ventral pons occupying > 75% of the cross-sectional area of the pons on at least one transverse T2-weighted image); 2) *Atypical DIPG* (atypical features including the following: predominantly pontine or pontobulbar location, eccentricity, disproportional extrapontine extension [s], < 75% cross-sectional involvement, well-defined margins, too much or no enhancement at all in post-contrast T1-weighted images, including subtraction T1-weighted pre- and post-contrast images, or dorsal exophytism suggesting tegmental origin); and 3) *Non-DIPG* (the tumor epicenter was considered to be extrapontine). For further details of the classification, see Supplementary Table 2.

### Statistical analyses

The median values; interquartile ranges; and range, count, and frequency measures were summarized by descriptive statistics. Progression-free survival (PFS) was defined as the time from diagnosis to progression, i.e., to local failure, distant failure (including leptomeningeal metastasis), or death, whichever occurred first. Overall survival (OS) was defined as the time from the date of diagnosis to death from any cause. Patients who did not experience an event were censored at the last follow-up date. Probability estimates of OS were calculated by the Kaplan-Meier (KM) method and compared using the log-rank test. Univariable analysis comparing aDIPG and tDIPG was performed using Fisher’s exact test or the Wilcoxon rank-sum test. A Cox proportional hazards model was used to identify imaging and clinicopathologic predictors of PFS and OS distributions for patients with aDIPG. Covariates with significant association at the *P* < 0.05 level were considered for inclusion in the multivariable analysis. Given the limited sample size, one covariate in addition to H3K27M status was evaluated in the model. Risk estimates, estimated by hazard ratios (HRs) and *P* values, and 95% confidence intervals were reported. Statistical analyses were performed using SAS version 9.4 or R version 3.1.3. A two-sided significance level of *P* < 0.05 was considered to indicate statistical significance.

## Results

### Clinicoradiographic features and surgical morbidity

**Fig. 1:**
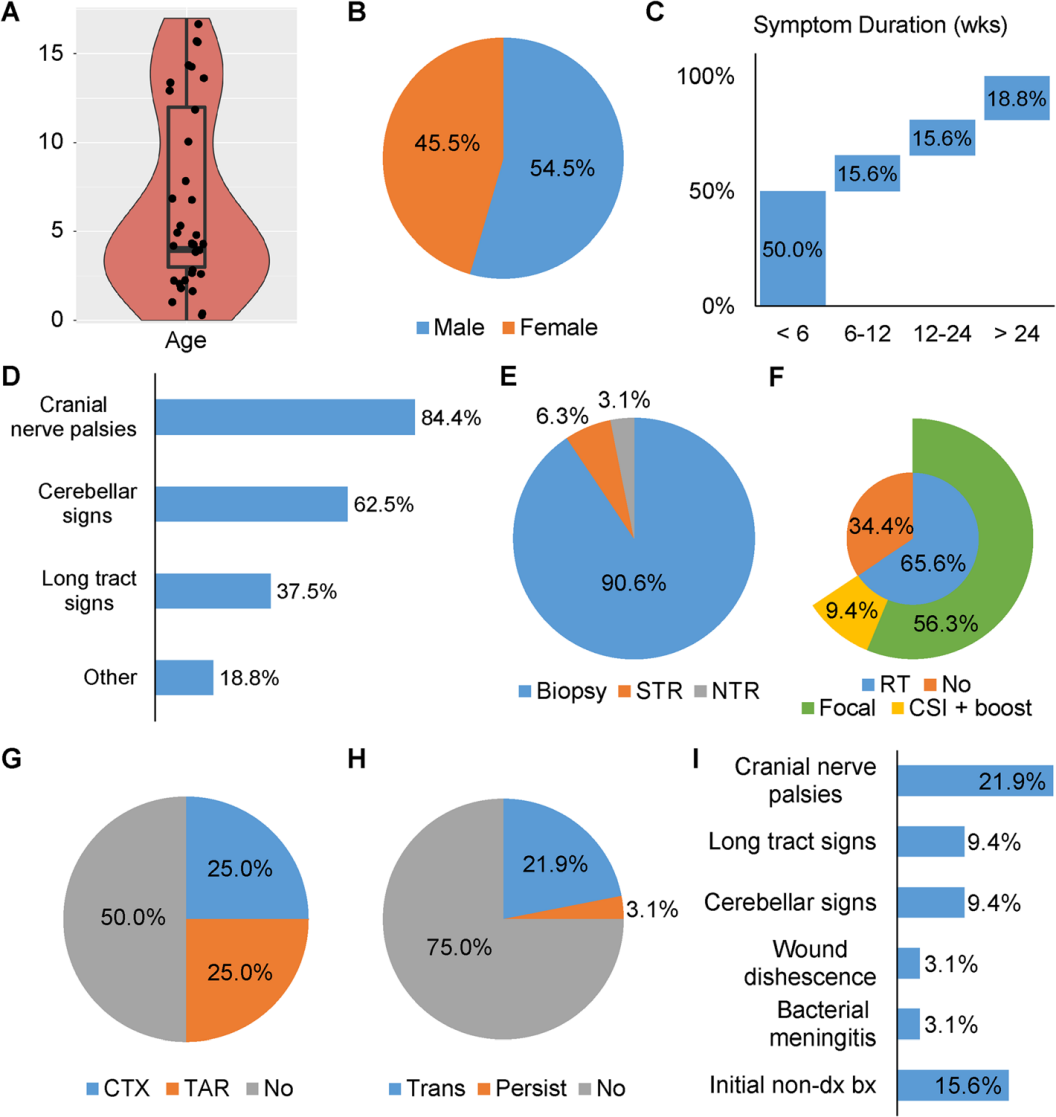
Clinical features of atypical DIPG (aDIPG). There was a bimodal age distribution of patients with aDIPG (**a**), with a slight male predilection (**b**). Fifty percent of patients had a symptom duration longer than 6 weeks at presentation (**c**). The most common presenting symptoms were cranial nerve palsies (**d**). Diagnostic surgical procedures performed are shown in (**e**) and treatment details are shown in (**f**) and (**g**). Most patients experienced no or only transient complications after surgery (**h**). Cranial nerve palsies were the most common complication after surgery (**i**). CSI, craniospinal irradiation; CTX, cytotoxic therapy; NTR, near-total resection; Persist: persistent; RT, radiation therapy; STR, subtotal resection; TAR, targeted therapy; Trans, transient

A total of 33 patients with a brainstem tumor diagnosed clinically as aDIPG at initial presentation and treated at our institution were evaluated. The clinical features of these patients are summarized in Fig. 1. There was a bimodal age distribution (Fig. 1a) and a slight male predilection (Fig. 1b). The median age was 4 years, although eight patients (24.2%) were older than 12 years. Fifty percent of the patients had a symptom duration longer than 6 weeks (median, 4 weeks; interquartile range [IQR], 2–24 weeks) (Fig. 1c). Cranial nerve palsies were the most common presenting symptoms, followed by cerebellar signs (Fig. 1d). Diagnostic biopsy was the most commonly performed surgical procedure (Fig. 1e). In contrast to most patients with tDIPG, only 65.6% of patients with aDIPG received radiation therapy (Fig. 1f). Amongst the focally irradiated patients, volumetric planning was uniformly applied with a prescribed total dose of 54Gy. Three patients were treated with craniospinal irradiation. Half of the patients received cytotoxic or targeted chemotherapy (Fig. 1g). Diagnostic surgical procedures were performed in 32 of 33 patients and were well tolerated, with 31 of the patients (96.9%) having no or only transient complications after surgery (Fig. 1h) and only one patient (3.1%) having persistent neurologic deficits. Transient neurologic deficits were observed in seven of the 32 patients (21.9%), with a median duration of 15.0 days (range, 5–30 days). Of these seven patients, four were started on or had an increase in dexamethasone, with a median starting dose of 3 mg/day (range, 2–4 mg/day) for a median duration of 21.5 days (range, 14–32 days). Worsening or new cranial nerve palsies, particularly facial palsies, were the most common post-surgical deficit (Fig. 1i). Five of the 32 patients (15.6%) had an initial non-diagnostic biopsy (Fig. 1i). There was no surgery-related death.

**Fig. 2:**
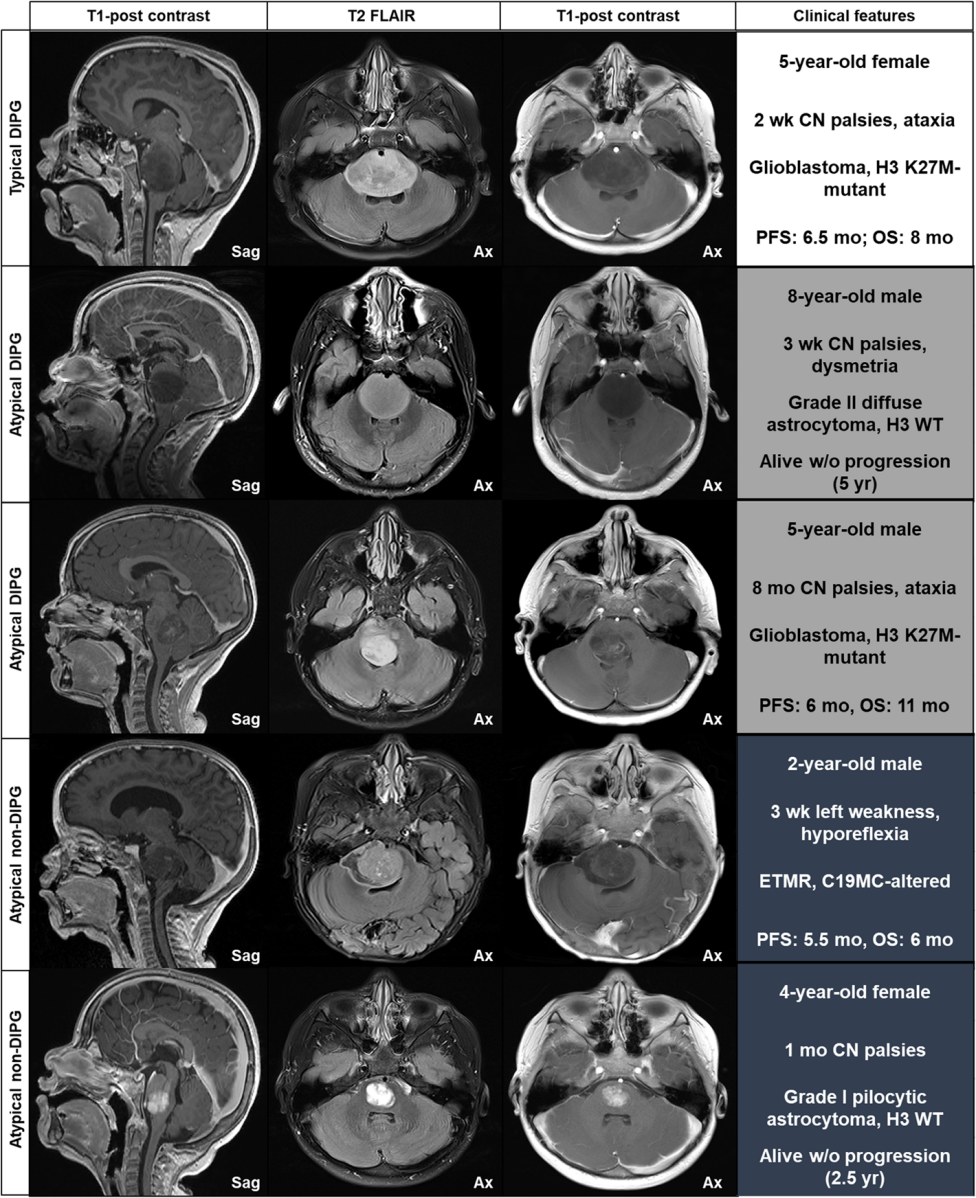
Imaging features of DIPG. The DIPG type is indicated at the left of each row, and the MRI sequence is indicated in the column headers. Clinical features, including patient age and sex; type and duration of neurologic symptoms; pathology; and outcome, with follow-up time indicated in parenthesis for living patients, are shown in the right column. Atypical imaging features included: 2nd row, lack of intralesional inhomogeneity and well-defined margins; 3rd row, tegmental epicenter with dorsal exophytism; 4th row, well-defined margins with diffusion restriction (not shown); 5th row, small focal tumor with uniform avid enhancement. Sag, sagittal; Ax, axial; CN, cranial nerve; PFS, progression-free survival; OS, overall survival; ETMR, embryonal tumor with multilayered rosettes; w/o, without

Preoperative diagnostic MR images were available for review for all patients, including the aDIPG and tDIPG cohorts. The frequencies of MRI features across the aDIPG cohort are summarized in Supplementary Table 3. Except for extension into the medulla, no other evaluated imaging feature was observed in more than 50% of aDIPG patients. Representative images of selected patients and the associated clinical, pathologic, and outcome characteristics are shown in Fig. 2.

### Histopathologic evaluation

**Fig. 3:**
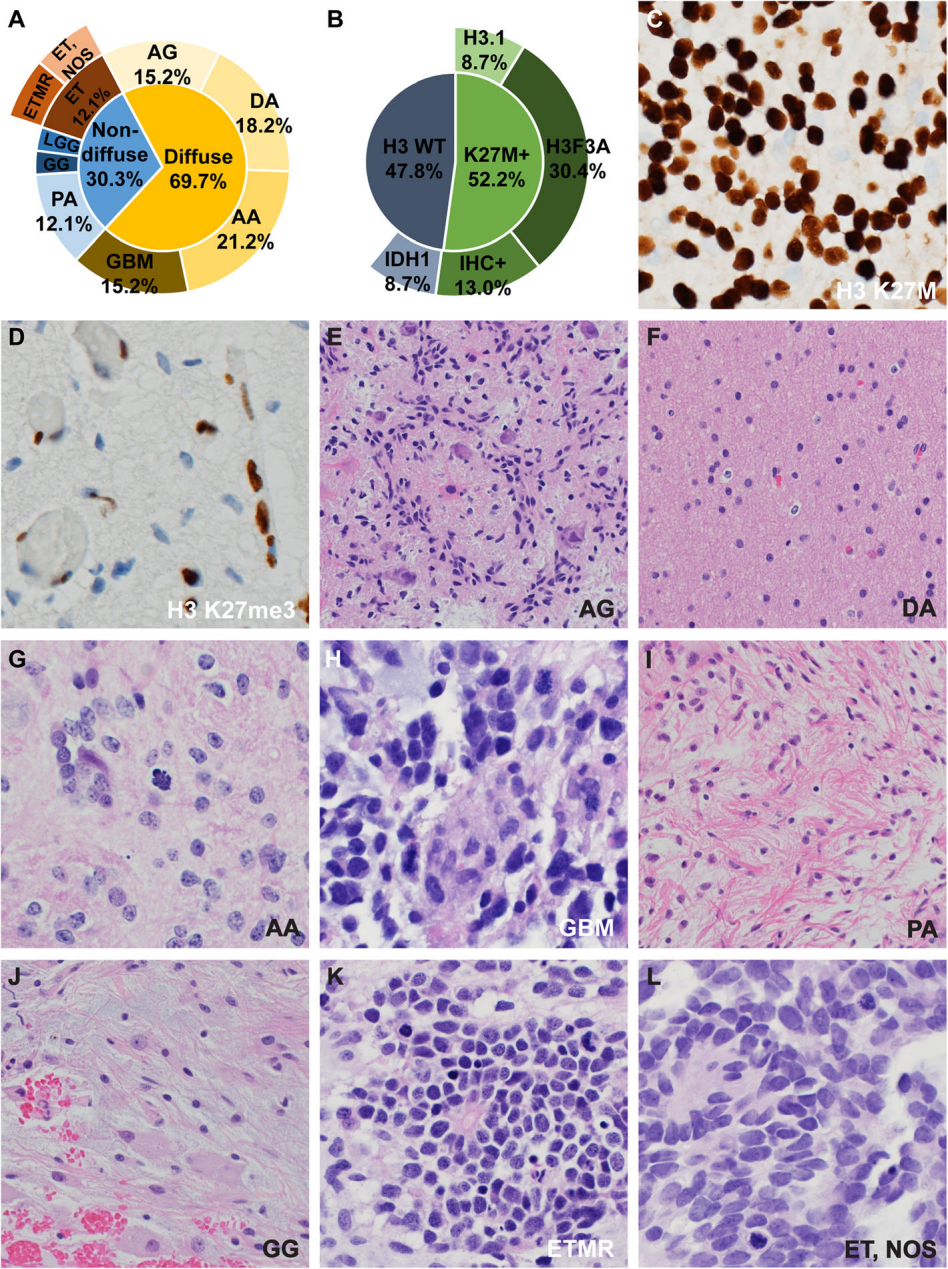
Histopathologic findings of atypical DIPG (aDIPG). A wide range of disease entities was identified in aDIPG (**a**). Only slightly more than half of the diffuse aDIPG harbored a histone H3 K27M mutation (**b**). Tissue sections immunostained for histone H3 K27M–mutant protein and trimethylation of the H3 K27 residue are shown in (**c**) and (**d**), respectively. The remaining panels are representative images of identified entities, including angiocentric glioma (AG) (**e**); diffuse astrocytoma (DA) (**f**); anaplastic astrocytoma (AA) (**g**); glioblastoma (GBM) (**h**); pilocytic astrocytoma (PA) (**i**); ganglioglioma (GG) (**j**); C19MC-altered embryonal tumor with multilayered rosettes (ETMR) (**k**); and CNS embryonal tumor, not otherwise specified (ET, NOS) (**l**)

Disease entities identified in the aDIPG cohort after a central review of the morphologic, immunophenotypic, and molecular findings are summarized in Fig. 3 and Supplementary Table 6. The clinical diagnosis of aDIPG encompassed a wide range of pathology. Twenty-three of the 33 tumors (69.7%) demonstrated a diffusely infiltrating growth pattern, and 10 (30.3%) showed compact (non-infiltrative) growth (Fig. 3a). No tumors with a non-infiltrative growth harbored a histone H3 K27M mutation. Only slightly more than half of the infiltrating tumors (52.2%) had a histone H3 K27M mutation (Fig. 3b–d). Seven cases with *H3F3A* mutations and two cases with *HIST1H3B* mutations were confirmed by sequencing. Two cases with *IDH1* mutations were identified; one with *IDH1* R132G and the other with *IDH1* R132C. No tumors in the aDIPG cohort harbored a *BRAF* V600E mutation. Entities that diffusely infiltrated the pontine parenchyma included *MYB*-rearranged angiocentric glioma (WHO grade I, all H3-wildtype, 5 cases [15.2%]) (Fig. 3e), diffuse astrocytoma (WHO grade II, 6 cases [18.2%]) (Fig. 3f), anaplastic astrocytoma (WHO grade III, 7 cases [21.2%]) (Fig. 3g), and glioblastoma (WHO grade IV, 5 cases [15.2%]) (Fig. 3h). Entities that showed non-infiltrative growth included pilocytic astrocytoma with *KIAA1549–BRAF* fusion (WHO grade I, 4 cases [12.1%]) (Fig. 3i), one *BRAF*-wildtype ganglioglioma (WHO grade I) (Fig. 3j), two C19MC-altered embryonal tumors with multilayered rosettes (WHO grade IV) (Fig. 3k), two CNS embryonal tumors, not otherwise specified (WHO grade IV) (Fig. 3l), and one low-grade glioma that demonstrated no alterations in histone H3, *IDH1*/*IDH2*, *MYB*, *BRAF*, *TP53*, or *ATRX*.

### Methylome profiling

**Fig. 4:**
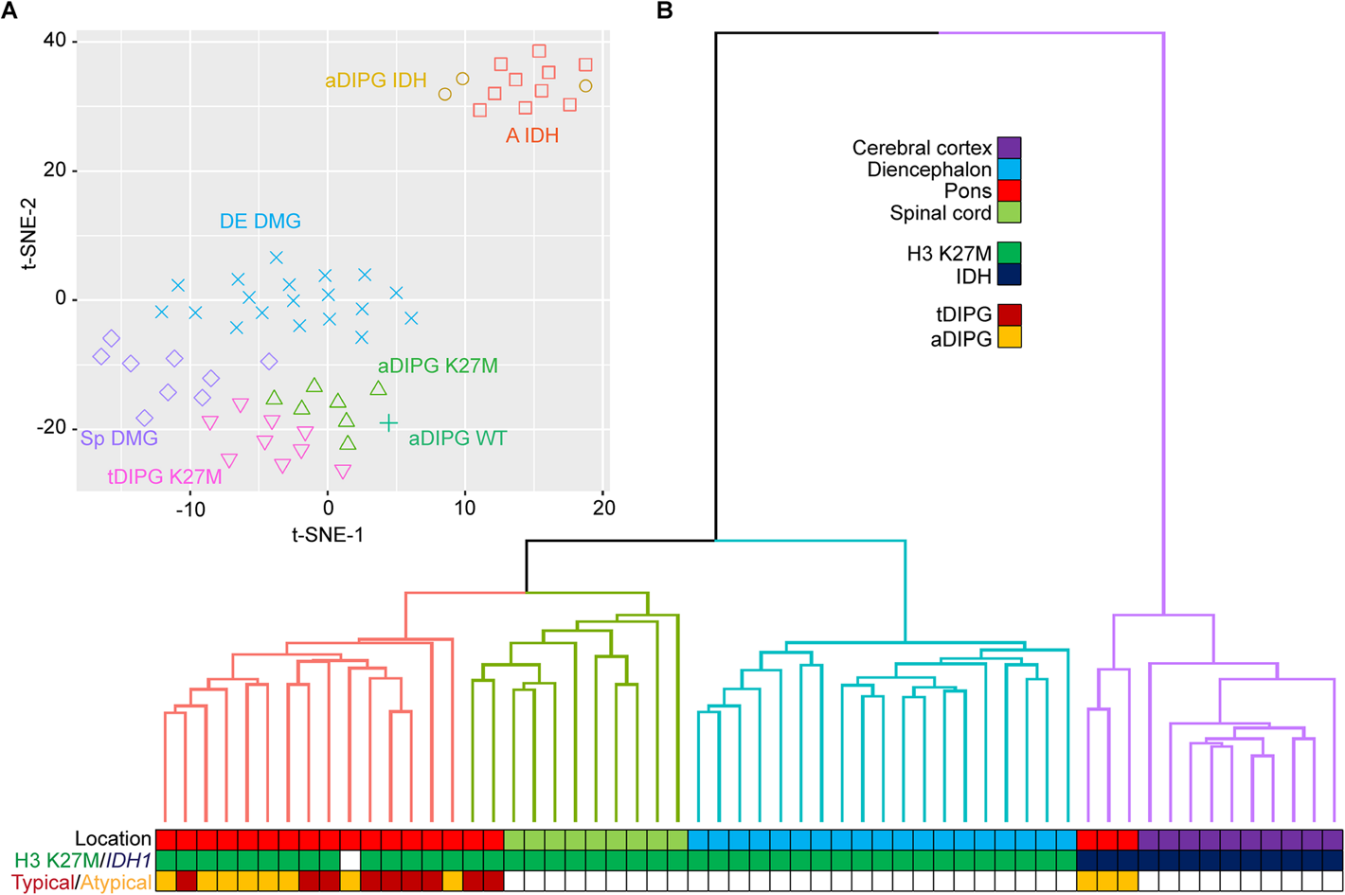
A t-distributed stochastic neighbor embedding (t-SNE) plot (**a**) and unsupervised cluster analysis (**b**) of methylome profiles of diffuse atypical DIPG (aDIPG). The methylation profiles of H3 K27M–mutant and IDH-mutant aDIPG formed distinct clusters with their respective typical and cerebral cortical counterparts

To further characterize diffusely infiltrating pontine tumors with histone H3 K27M or *IDH1* R132 mutations diagnosed clinically as “aDIPG”, their genome-wide DNA methylome profiles were compared with those of H3 K27M–mutant tDIPG, DMG of the diencephalon or spinal cord, and IDH-mutant astrocytomas of the cerebral cortex by t-SNE and unsupervised cluster analyses. As shown in Fig. 4, Supplementary Figure 1, and Supplementary Figure 2, the methylome profiles of H3 K27M–mutant aDIPG and tDIPG showed no significant differences but formed a group distinct from DMG of the diencephalon and spinal cord, suggesting that there are differences in the underlying biology of these tumors. In contrast, the methylome profiles of IDH-mutant aDIPG clustered together with those of cerebral cortical IDH-mutant astrocytomas. The methylome profile of one H3/IDH-wildtype anaplastic astrocytoma clustered together with H3 K27M-mutant tDIPG and aDIPG, indicating that histone H3 K27M mutation may not be required to generate a DMG-like methylome profile. The H3/IDH-wildtype anaplastic astrocytoma harbored I596T and E610K double mutations (in *cis*) in *TCF12*, identified by WGS, WES, and RNA-seq. Similar double mutations (*TCF12* R423*/E610K) were also identified in one H3 K27M–mutant aDIPG by WGS, WES, and RNA-seq. These alterations may compromise the function of TCF12. Similar alterations have also been seen in anaplastic oligodendrogliomas [22]. Pontine *MYB*-altered gliomas presented clinically as aDIPG clustered together with *MYB*-altered gliomas of other sites (Supplementary Figure 2), as previously described [17].

### Variables prognostic of outcome and associated with H3 K27M status

**Fig. 5:**
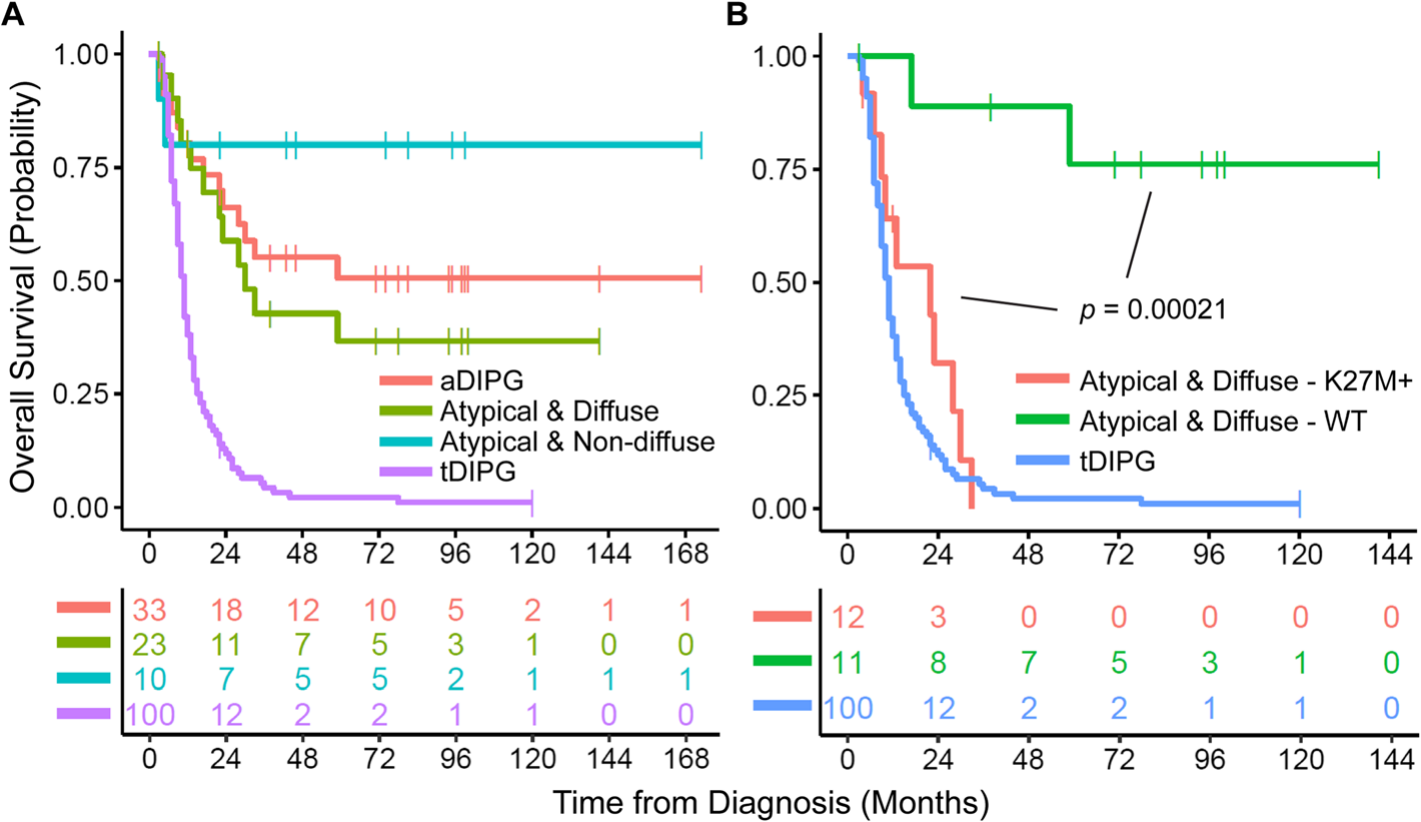
Overall survival (OS) of the atypical DIPG (aDIPG) cohort (**a**) compared with that of a contemporary typical DIPG (tDIPG) cohort. There was no significant difference in OS among the subgroups of patients with aDIPG, whereas the OS of patients with tDIPG was significantly worse (*P* < 0.00001). Histone H3 K27M mutation status was the major determinant of OS in diffuse aDIPG (**b**). There was no significant difference in OS between patients with H3 K27M–mutant aDIPG and tDIPG

Univariable analysis revealed that ring enhancement, high WHO grade (III or IV), H3 K27M mutation, and *TP53* mutation were adverse prognostic indicators for OS in aDIPG (Supplementary Table 4). Multivariable analysis further revealed that H3 K27M mutation was the most significant adverse prognostic factor of OS. As a group, patients with the clinical diagnosis of aDIPG had favorable OS when compared with those with tDIPG (Fig. 5a) due to the presence of low-grade tumors in a significant portion of the patients. Patients with non-diffuse aDIPG had better survival than did those with diffuse aDIPG, which is to be expected based on the spectrum of histopathology: a significant portion of non-diffuse aDIPG were WHO grade I tumors (Fig. 3a). Consistent with the multivariable analysis, histone H3 K27M mutation emerged as the major adverse prognosticator for diffuse aDIPG (Fig. 5b), as patients with diffuse aDIPG harboring a histone H3 K27M mutation had similar OS to those with tDIPG.

There was no significant difference in the clinical or imaging parameters for aDIPG with and without histone H3 K27M mutation (Supplementary Table 5). However, aDIPG without histone H3 K27M mutation presented with several statistically significant distinct clinicoradiographic features compared to tDIPG. Clinically, aDIPG without histone H3 K27M mutation were more likely than tDIPG to present without pyramidal tract symptoms or cranial nerve palsies. Radiographically, aDIPG without histone H3 K27M mutation were smaller, more likely to be eccentric in the pons, more likely to show exophytic growth, less likely to have ring enhancement, and less likely to involve the mesencephalon compared to tDIPG (Table 1).

**Table 1:** Univariable analysis comparing clinical and radiographic variables between atypical DIPG (aDIPG) without an H3 K27M mutation and typical DIPG (tDIPG)

**Characteristic**	**aDIPG without K27M mutation (*n*** **= 21)**	**tDIPG (*n*** **= 100)**	***P***
Clinical			
Age @ diagnosis (years), median (IQR)	4.8 (2.5,7.6)	6.2 (4.2,8.4)	0.09
Sex, no. (%)			
Male	12 (57%)	45 (45%)	0.34
Female	9 (43%)	55 (55%)	
Race, no. (%)			
Black	5 (24%)	18 (18%)	0.31
White	12 (57%)	72 (72%)	
Other	4 (19%)	10 (10%)	
Symptom duration (mo), median (IQR)	1.0 (0.5,6.0)	1.0 (0.5,2.0)	0.52
**Cranial nerve palsy, no. (%)**			
**Yes**	**17 (81%)**	**95 (95%)**	**0.05**
**No**	**4 (19%)**	**5 (5%)**	
**Pyramidal tract symptoms, no. (%)**			
**Yes**	**8 (38%)**	**70 (70%)**	**0.01**
**No**	**13 (62%)**	**30 (30%)**	
Cerebellar symptoms, no. (%)			
Yes	13 (62%)	78 (78%)	0.16
No	8 (38%)	22 (22%)	
CSF diversion, no. (%)			
Yes	3 (14%)	17 (17%)	1.00
No	18 (86%)	83 (83%)	
**Systemic therapy @ diagnosis, no. (%)**			
**Yes** **No**	**11 (52%)** **10 (48%)**	**92 (92%)** **8 (8%)**	**<.0001**
*Radiologic*			
**Tumor size (mL), median (IQR)**	**20.5 (16.0,37.5)**	**38.8 (27.9,51.4)**	**0.002**
**Ring enhancement @ diagnosis, no. (%)**			
**Yes**	**1 (5%)**	**42 (42%)**	**0.001**
**No**	**20 (95%)**	**58 (58%)**	
**Growth in mesencephalon, no. (%)**			
**Yes**	**6 (29%)**	**86 (86%)**	**<.0001**
**No**	**15 (71%)**	**14 (14%)**	
Growth in medulla, no. (%)			
Yes	13 (62%)	74 (74%)	0.29
No	8 (38%)	26 (26%)	
Growth in middle cerebellar peduncle, no. (%)			
Yes	10 (48%)	67 (67%)	0.13
No	11 (52%)	33 (33%)	
Tumor margin, no. (%)			
Ill-defined	14 (67%)	71 (71%)	0.79
Well-defined	7 (33%)	29 (29%)	
**Eccentricity within pons, no. (%)**			
**Yes**	**9 (43%)**	**20 (20%)**	**0.05**
**No**	**12 (57%)**	**80 (80%)**	
**Extrapontine extension, no. (%)**			
**Yes**	**7 (33%)**	**11 (11%)**	**0.02**
**No**	**14 (67%)**	**89 (89%)**	

Abbreviations: *IQR* interquartile range, *WHO* World Health Organization

### Neuro-imaging review

Given inconsistencies in defining DIPG as typical or atypical based on MRI [3, 23], diagnostic MR images of the aDIPG cohort were re-evaluated by a neuroradiologist (Z.P.) who was blinded to the histologic diagnoses or clinical characteristics of the cohort. When evaluated using a subjective classification scheme of conventional MRI features, only 17/33 cases (52%) were considered to represent aDIPG based on imaging features alone, with the remaining cases being considered tDIPG (8/33, 24%) or non-DIPGs with extrapontine epicenters (8/33, 24%) (Supplementary Table 2). Of the cases considered aDIPG upon re-review, 4/17 tumors exhibited features of embryonal tumors based on their diffusion-weighted imaging features, 4 tumors exhibited features characteristic of pilocytic astrocytoma (e.g., avid enhancement and well-defined margins), and 3 tumors harbored large, non-petechial intratumoral hemorrhages consistent with glioblastoma. The remaining 6/17 cases were considered atypical based on considerable extrapontine extension (3), eccentric ponto-bulbar location (2) and small tumor size (< 50% involvement of the pons) (1).

Except for embryonal tumors which were perfectly correlated, classification by imaging correlated poorly with histopathologic diagnosis (Supplementary Table 2). Although H3 K27M–mutant DMG was the most common histology of cases considered to represent tDIPG (50%) upon re-review, diffuse astrocytomas without H3 mutation (25%) and grade I gliomas (25%) were also observed in this group. Conversely, H3 K27M–mutant DMG was observed in 35% of aDIPG and in 25% of cases considered to have an epicenter outside the pons.

## Discussion

Our study represents the first comprehensive analysis of the clinical, imaging, histopathologic, and molecular features of pediatric pontine tumors with radiographic characteristics deviating from those of tDIPG at initial diagnostic workup. We found diverse histologies within this clinical entity ‘aDIPG’, including grade I gliomas (pilocytic and angiocentric gliomas) and gangliogliomas, grade II to IV diffuse astrocytic tumors with or without the H3 K27M mutation, and embryonal tumors. The frequency of histone H3 K27M mutation in aDIPG (36.4%) was significantly lower than that in tDIPG (approximately 80%) [24]. The surgical morbidity associated with procedures that facilitated this therapy and prognosis-changing differential diagnosis was modest, with a similar rate of persistent neurologic deficits being observed in patients with presumed tDIPG [25].

Consistent with reports of midline high-grade glioma [13, 26, 27], histone H3 K27M mutation was most prognostic of survival, with outcomes of aDIPG harboring an H3 K27M mutation being similar to those of tDIPG. Within the limitation of the small sample sizes, we identified no clinicoradiographic features of aDIPG that correlated with H3 K27M status. Nevertheless, several clinical and imaging features differed significantly between aDIPG without H3 K27M mutation and tDIPG, and these differences may have utility in defining patients who may most benefit from diagnostic biopsy. Similar to clinicoradiographic features, the methylome profiles of H3 K27M–mutant aDIPG did not differ significantly from those of H3 K27M–mutant tDIPG. However, both aDIPG and tDIPG with H3 K27M mutation appear to be epigenetically distinct from DMG of the diencephalon and spinal cord. These findings suggest that H3 K27M–mutant DMG presenting as tDIPG or aDIPG are histologically, genetically, and epigenetically very similar, as reflected by their similar survival outcomes.

Despite the established criteria for the radiographic diagnosis of DIPG, most studies have demonstrated a “tail” to the Kaplan-Meier survival curve, with 5–10% of patients surviving beyond 2 years and a 5-year survival of 2–3% [1, 2, 28]. Without pathologic assessment in most cases, this prolonged survival has been presumed, at least in part, to be the result of misdiagnosis of underlying histopathology. This may reflect the subjectivity and inconsistencies of interpreting radiologic features [3, 23]. Indeed, in a review of published reports of very-long-term survivors of DIPG (e.g., those surviving more than 5 years after diagnosis), seven of 38 cases were considered to represent aDIPG upon re-review, despite the very similar radiographic diagnostic criteria used to establish a diagnosis of DIPG in the initial studies [1]. This discrepancy may also result from the lack or inconsistent use of clinical diagnostic features, including neurologic symptom type and duration, characteristics that have been consistently associated with survival [1, 2]. Furthermore, given the historic practice pattern of a clinical diagnosis of DIPG, defining the “true” histopathology of DIPG is confounded. Many clinical trials, both completed and ongoing, allow for disparate histologies, including anaplastic mixed glioma, gliosarcoma, and fibrillary astrocytoma [29, 30]. There is a particular controversy surrounding the optimal treatment and prognosis of WHO grade II diffuse astrocytomas of the pons that lack the H3 K27M mutation.

Understanding the limitations of reproducibly defining tDIPG or variants thereof, this study was restricted to patients with a histopathologic diagnosis obtained in the setting of baseline MR imaging, with or without clinical features, interpreted as inconsistent with a diagnosis of DIPG. We found that, although the proportion of patients with tumors that might be considered distinct from DIPG was significantly higher than that observed in historic DIPG trials based on long-term survival (approximately 50% vs. < 10%), grade II–IV diffuse astrocytoma with or without H3 K27M mutation was observed in more than half of the cases. Additionally, if the diagnosis of DIPG is restricted to patients that fulfill the current WHO diagnostic criteria for DMG, approximately one-third of aDIPG would be classified as such. Our blinded neuroimaging review further demonstrates the difficulty of ascertaining a diagnosis of DIPG based on imaging alone. Approximately a quarter of the cases initially described clinically as aDIPG were considered to be similar to tDIPG upon re-review, whereas diffuse gliomas harboring an H3 K27M mutation, the defining lesion of DIPG, were observed in 60% of the remaining cases. This once again argues for biopsy of aDIPG and suggests that biopsy of tDIPG should also be considered at experienced centers.

A principle concern regarding biopsy for presumed DIPG is the associated surgical morbidity and mortality. Several recent observational studies have suggested that brainstem biopsy in pediatric patients is relatively safe, with a metanalysis of 735 patients demonstrating a weighted average proportion of 6.7% for overall morbidity, 0.6% for permanent morbidity, and 0.6% for mortality [31]. A prospective phase II clinical trial in which biopsy of presumed DIPG was mandated reported no biopsy-attributed deaths, and only one patient of 50 (2%) experienced a persistent neurologic deficit after biopsy [32], with similar outcomes being observed in two recent prospective studies mandating biopsy [25, 33]. The risks of biopsy may vary by lesion location, among other host, tumor, and technical factors, and the safety profile of biopsy for patients with aDIPG has not been specifically reported. Our study showed that diagnostic surgical procedures for aDIPG were generally well tolerated, as the vast majority of patients experienced no or only transient neurologic deficits. Persistent neurologic deficits were seen in one of 32 patients (3.1%), which is in the range reported for tDIPG (0–5%) [25, 31–34] and for adults with primarily supratentorial glioblastoma after surgical resection (3.8%) [10], suggesting that the frequency of this adverse outcome may not necessarily differ by tumor location. However, persistent surgical morbidity can be considerable. Our patient experienced persistent and complete right facial palsy complicated by exposure keratitis with subsequent right tarsorrhaphy. Pathology revealed pilocytic astrocytoma, and radiation therapy was deferred, highlighting the complexities involved when considering biopsy for these patients.

On univariable analysis of clinicopathologic features, we found that ring enhancement on baseline MRI, high tumor grade, and H3 K27M and *TP53* mutations were associated with worse survival in patients with aDIPG. Multivariable models demonstrated the H3 K27M mutation to be the most significant prognostic factor for survival. Consistent with this analysis, patients with aDIPG harboring an H3 K27M mutation had similar survival to a contemporary cohort of patients with tDIPG treated at our institution. In an attempt to identify factors specific to patients with aDIPG that lack the K27M mutation and who may most benefit from a diagnostic surgical procedure, univariable analysis revealed significant differences in the presenting neurologic symptoms and imaging features of patients with aDIPG without H3 K27M mutation and patients with tDIPG. These differences included cranial nerve palsies and pyramidal tract signs, as well as tumor size, ring enhancement, growth in the mesencephalon, eccentricity within the pons, and extrapontine extension. Given the clinical utility of noninvasive methods to predict H3 K27M mutation status, there have been recent advances in radiogenomic modeling of baseline conventional MR images in patients with midline glioma [24, 35]. In the future, when larger datasets of DIPG with pathologic assessment may be developed, this methodology could be useful for defining additional radiographic features predictive of H3 status specifically in patients with presumed DIPG and, ultimately, may enable radiogenomic pipelines that facilitate real-time clinical translation of these findings.

## Supplementary information

**Additional file 1: Figure S1.** Heatmap of unsupervised cluster analysis using the 5000 most variable probes.

**Additional file 2: Figure S2.** t-SNE analysis of atypical DIPG with a reference series of 265 samples of 20 CNS tumor entities and normal tissue from the cerebellum and pons. A IDH: IDH-mutant astrocytoma. CB: Cerebellum. CBPA: Cerebellar pilocytic astrocytoma. DE DMG: Diencephalic diffuse midline glioma. DG MYB: Diffuse glioma with *MYB* alteration. DNET: Dysembryoplastic neuroepithelial tumor. GG: Ganglioglioma. HTPA: Hypothalamic pilocytic astrocytoma. O IDH: IDH-mutant and 1p/19q-codeleted oligodendroglioma. RGNT: Rosette-forming glioneuronal tumor. sDNET: Septal dysembryoplastic neuroepithelial tumor. SEGA: Subependymal giant cell astrocytoma. Sp DMG: Spinal cord diffuse midline glioma. TG: Tectal glioma.

**Additional file 3: Table S1.** Primer sequences.

**Additional file 4: Table S2.** Blinded re-review of diagnostic MR imaging of the 33 aDIPG cases and their corresponding histopathologic diagnosis.

**Additional file 5: Table S3.** Preoperative MR imaging features of the 33 atypical DIPG in the study cohort.

**Additional file 6: Table S4.** Univariable and multivariable Cox proportional analysis of overall survival of patients with atypical DIPG.

**Additional file 7: Table S5.** Univariable analysis comparing clinical and radiographic variables between atypical DIPG without and with H3 K27M mutation.

**Additional file 8 Table S6.** Molecular analyses and findings of the study cohort.
