# Supplementary figures and images for: Clinical, imaging, and molecular analysis of pediatric pontine tumors lacking characteristic imaging features of DIPG
Source: Acta Neuropathol Commun. 2020 Apr 23;8:57. doi: 10.1186/s40478-020-00930-9 (PMC7181591; doi:10.1186/s40478-020-00930-9)

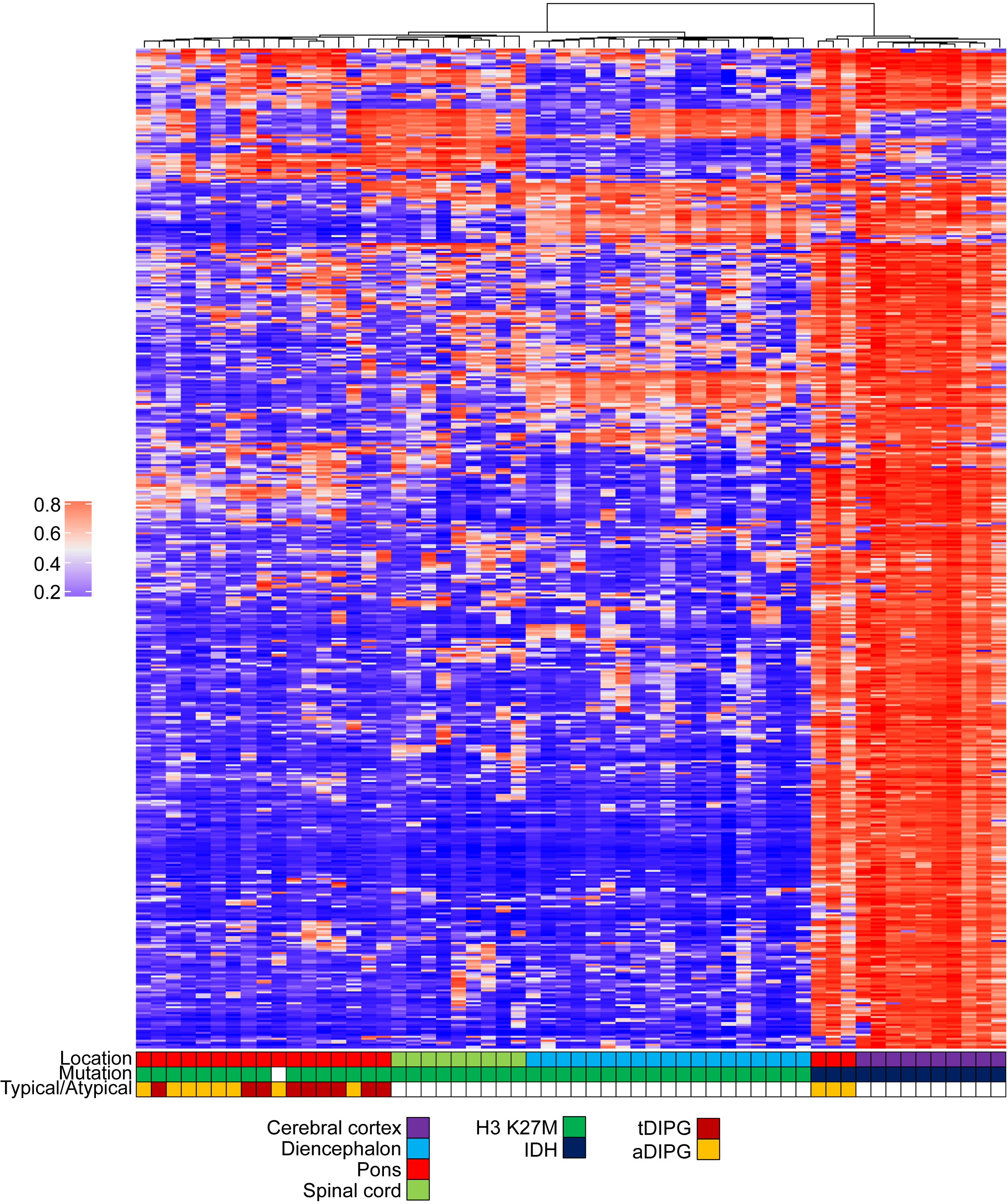

Supplement: Supplementary file 1 — Additional file 1: Figure S1. Heatmap of unsupervised cluster analysis using the 5000 most variable probes. [file 40478_2020_930_MOESM1_ESM.tif]

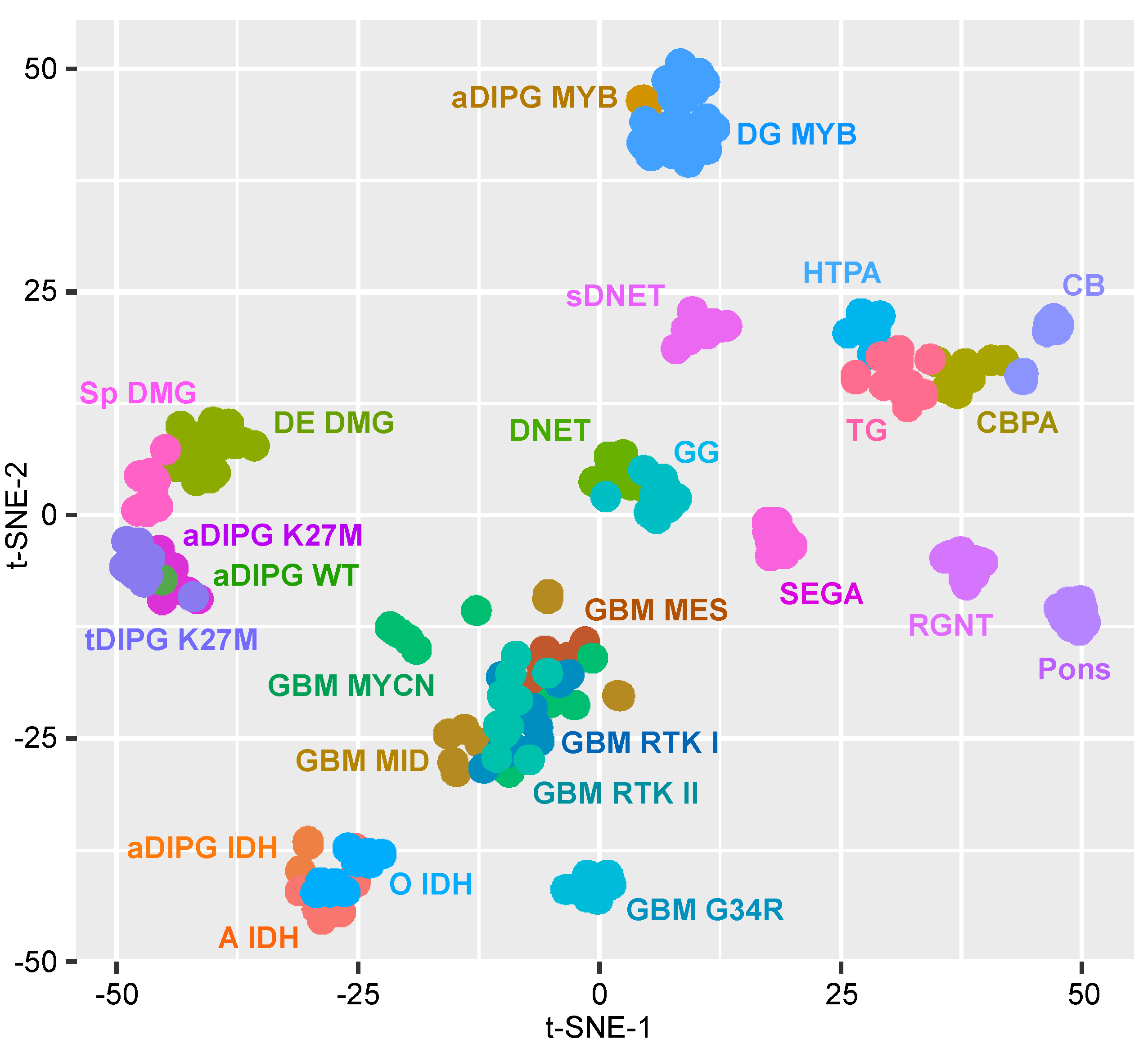

Supplement: Supplementary file 2 — Additional file 2: Figure S2. t-SNE analysis of atypical DIPG with a reference series of 265 samples of 20 CNS tumor entities and normal tissue from the cerebellum and pons. A IDH: IDH-mutant astrocytoma. CB: Cerebellum. CBPA: Cerebellar pilocytic astrocytoma. DE DMG: Diencephalic diffuse midline glioma. DG MYB: Diffuse glioma with MYB alteration. DNET: Dysembryoplastic neuroepithelial tumor. GG: Ganglioglioma. HTPA: Hypothalamic pilocytic astrocytoma. O IDH: IDH-mutant and 1p/19q-codeleted oligodendroglioma. RGNT: Rosette-forming glioneuronal tumor. sDNET: Septal dysembryoplastic neuroepithelial tumor. SEGA: Subependymal giant cell astrocytoma. Sp DMG: Spinal cord diffuse midline glioma. TG: Tectal glioma. [file 40478_2020_930_MOESM2_ESM.tif]
