# Supplementary material for: Clinical, imaging, and molecular analysis of pediatric pontine tumors lacking characteristic imaging features of DIPG
Source: Acta Neuropathol Commun. 2020 Apr 23;8:57. doi: 10.1186/s40478-020-00930-9 (PMC7181591; doi:10.1186/s40478-020-00930-9)
Supplement: Supplementary file 3 — Additional file 3: Table S1. Primer sequences. [file 40478_2020_930_MOESM3_ESM.docx]

**Supplementary Table 1.** Primer sequences.

| Target | Primer | Sequence (5′–3′) |
| --- | --- | --- |
| *H3F3A* K27 | H3F3A-4-5F | ATGCTGGTAGGTAAGTAAGG |
|  | H3F3A-4-3R | AACGATGAGGTTTCTTCACC |
|  | H3F3A-5-5F | TAGGTAAGTAAGGAGGTCTC |
|  | H3F3A-5-3R | TTTCTTCACCCCTCCAGTAG |
|  | H3F3A-6-5F | AGGTCTCTGTACCATGGCTC |
| *HIST1H3B* K27 | H3.1B-3-5F | TCTCTGCAGGCAAGCTTTTC |
|  | H3.1B-4-3R | AACGGTGAGGCTTTTTCACG |
|  | H3.1B-4-5F | TTCTGTGGTTTTGCCATGGC |
|  | H3.1B-5-3R | TTTTTCACGCCGCCGGTAG |
|  | H3.1B-5-5F | TGGCTCGTACTAAACAGACAGC |
| *HIST1H3C* K27 | H3.1C-3-5F | AGGCCACTTGCTCTCAGTTC |
|  | H3.1C-4-3R | TAGCGATGAGGTTTCTTCACG |
|  | H3.1C-4-5F | ACACTTTTGTGTGTGCTCTC |
|  | H3.1C-5-3R | ATGAGGTTTCTTCACGCCAC |
|  | H3.1C-5-5F | ATTGCAAATGGCTCGTACGAAG |
| *HIST1H3A* K27 | H3.1A-1-5F | TTGGGTGTTCCGCTGTGCTG |
|  | H3.1A-1-3R | TTTTTCACGCCGCCGGTG |
|  | H3.1A-2-5F | TGCTGTTTTTCCGTCATGGC |
|  | H3.1A-3-5F | ATGGCTCGCACTAAGCAAAC |
