# Supplementary material for: Clinical, imaging, and molecular analysis of pediatric pontine tumors lacking characteristic imaging features of DIPG
Source: Acta Neuropathol Commun. 2020 Apr 23;8:57. doi: 10.1186/s40478-020-00930-9 (PMC7181591; doi:10.1186/s40478-020-00930-9)
Supplement: Supplementary file 4 — Additional file 4: Table S2. Blinded re-review of diagnostic MR imaging of the 33 aDIPG cases and their corresponding histopathologic diagnosis. [file 40478_2020_930_MOESM4_ESM.docx]

**Supplementary Table 2.** Blinded re-review of diagnostic MR imaging of the 33 aDIPG cases and their corresponding histopathologic diagnosis.

| **Imaging pattern** | **Features** | **N (%)** | **Histopathologic diagnosis** |
| --- | --- | --- | --- |
| Typical DIPG | Intra-axial expansile lesion centered on the ventral pons occupying >75% of the cross-sectional area of the pons on at least one transverse T2-weighted image. If exhibiting exophytism, it is ventral, resulting in some degree of engulfment of the basilar artery. Extrapontine extensions are highly characteristic. Overt signal enhancement after intravenous contrast injection is common; however, this may be occult. | 8 (24%) |  |
| Subtype I | “Classic,” but no enhancement; alternative diagnosis unlikely | 2 (6%) | 1. Diffuse astrocytoma  2. Diffuse midline glioma, H3 K27M–mutant (glioblastoma) |
| Subtype II | T2 bright, better defined, no or minimal enhancement; alternative diagnosis unlikely | 6 (18%) | 1. Low-grade glioma, NOS 2. Pilocytic astrocytoma 3. Diffuse astrocytoma 4. Diffuse midline glioma, H3 K27M–mutant (anaplastic astrocytoma, 3 cases) |
| Atypical DIPG | Atypical features included any of the following: dominantly extrapontine location, eccentricity, disproportional extrapontine extension(s), <50% cross-sectional involvement, well-defined margins, too much or too little enhancement, dorsal exophytism | 17 (52%) |  |
| Subtype I | Small, limited tumor (<50%); alternative diagnosis unlikely | 1 (3%) | Diffuse midline glioma, H3 K27M–mutant (glioblastoma) |
| Subtype II | Pontine-centered, multifocal involvement, hemorrhagic; alternative diagnosis of higher-grade glioma possible | 3 (9%) | 1. Diffuse midline glioma, H3 K27M–mutant (glioblastoma)  2. Diffuse midline glioma, H3 K27M–mutant (diffuse astrocytoma)  3. Anaplastic astrocytoma |
| Subtype III | Eccentric morphology, ponto-bulbar-centered, involvement of the middle and inferior cerebellar peduncles; alternative diagnosis unlikely, yet may be considered non-DIPG based location alone | 5 (15%) | 1. Angiocentric glioma (2 cases)  2. Ganglioglioma  3. Diffuse midline glioma, H3 K27M–mutant (diffuse astrocytoma)  4. Anaplastic astrocytoma |
| Subtype IV | Pontine-centered, multifocal involvement, hemorrhagic; alternative diagnosis of higher-grade glioma possible | 3 (9%) | 1. Diffuse midline glioma, H3 K27M–mutant (glioblastoma)  2. Diffuse midline glioma, H3 K27M–mutant (diffuse astrocytoma)  3. Anaplastic astrocytoma |
| Subtype V | Pontine-centered, tegmental involvement, well-defined, more pronounced enhancement; alternative diagnosis of pilocytic astrocytoma possible | 1 (3%) | Diffuse midline glioma, H3 K27M–mutant (anaplastic astrocytoma) |
| Subtype VI | Ventrally located, ponto-bulbar involvement, more pronounced enhancement; alternative diagnosis of pilocytic astrocytoma possible | 3 (9%) | 1. Pilocytic astrocytoma (2 cases)  2. Diffuse midline glioma, H3 K27M–mutant (glioblastoma) |
| Subtype VII | Pontine-centered, well-defined; restricted diffusion; alternative diagnosis of embryonal tumor possible, yet may be considered non-DIPG based on location alone | 4 (12%) | 1. CNS embryonal tumor, NOS (2 cases)  2. Embryonal tumor with multilayer rosettes, C19MC-altered (2 cases) |
| Non-DIPG | Extrapontine epicenter | 8 (24%) |  |
| Subtype I | Bulbar-centered, inferior cerebellar peduncle and pontine tegmental extension, no or minimal enhancement; alternative diagnosis unlikely | 7 (21%) | 1. Angiocentric glioma (2 cases)  2. Diffuse astrocytoma (2 cases)  3. Pilocytic astrocytoma (2 cases)  4. Diffuse midline glioma, H3 K27M–mutant (glioblastoma) |
| Subtype II | Non-brainstem, extra-axial, cerebellopontine angle-centered; alternative diagnosis unlikely | 1 (3%) | Diffuse midline glioma, H3 K27M–mutant (anaplastic astrocytoma) |
