# Supplementary material for: Clinical, imaging, and molecular analysis of pediatric pontine tumors lacking characteristic imaging features of DIPG
Source: Acta Neuropathol Commun. 2020 Apr 23;8:57. doi: 10.1186/s40478-020-00930-9 (PMC7181591; doi:10.1186/s40478-020-00930-9)
Supplement: Supplementary file 5 — Additional file 5: Table S3. Preoperative MR imaging features of the 33 atypical DIPG in the study cohort. [file 40478_2020_930_MOESM5_ESM.docx]

**Supplementary Table 3.** Preoperative MR imaging features of the 33 atypical DIPG in the study cohort.

| **Baseline MRI feature** | **N (%)** |
| --- | --- |
| Eccentricity within pons | 16 (48.5) |
| Contrast enhancement | 14 (42.4) |
| Extrapontine extension | 10 (30.3) |
| Medullary extension | 19 (57.8) |
| Cerebellar peduncle extension | 14 (42.4) |
| Midbrain extension | 9 (27.3) |
| Well circumscribed | 9 (27.3) |
| Basilar artery encasement | 12 (36.4) |
| Tegmental sparing | 15 (45.5) |
| Hydrocephalus | 5 (15.2) |
