# Supplementary material for: Clinical, imaging, and molecular analysis of pediatric pontine tumors lacking characteristic imaging features of DIPG
Source: Acta Neuropathol Commun. 2020 Apr 23;8:57. doi: 10.1186/s40478-020-00930-9 (PMC7181591; doi:10.1186/s40478-020-00930-9)
Supplement: Supplementary file 6 — Additional file 6: Table S4. Univariable and multivariable Cox proportional analysis of overall survival of patients with atypical DIPG. [file 40478_2020_930_MOESM6_ESM.docx]

**Supplementary Table 4.** Univariable and multivariable Cox proportional analysis of overall survival of patients with atypical DIPG.

| ***Univariable analysis*** | | | |
| --- | --- | --- | --- |
| **Covariate** | ***P value*** | ***Hazard ratio (95% CI)*** |  |
| *Clinical* | | | |
| Age @ diagnosis (years) | 0.08 | 1.09 (0.99, 1.21) |  |
| Sex (female vs. male) | 0.94 | 1.04 (0.35, 3.10) |  |
| Race (%) | 0.19 |  |  |
| Black vs. white | 0.42 | 0.53 (0.11, 2.47) |  |
| Other vs. white | 0.14 | 3.25 (0.68, 15.54) |  |
| Symptom duration (mo) | 0.33 | 0.96 (0.88, 1.04) |  |
| Cranial nerve palsy (no vs. yes) | 0.57 | 1.45 (0.40, 5.29) |  |
| Pyramidal tract symptoms (no vs. yes) | 0.19 | 2.39 (0.66, 8.69) |  |
| Cerebellar symptoms (no vs. yes) | 0.25 | 1.91 (0.64, 5.76) |  |
| CSF diversion (no vs. yes) | 0.82 | 0.79 (0.10, 6.17) |  |
| Systemic therapy @ diagnosis (no vs. yes) | 0.93 | 1.05 (0.35, 3.14) |  |
| *Radiologic* | | | |
| Tumor size (mL) | 0.94 | 1.00 (0.96, 1.04) |  |
| **Ring enhancement @ diagnosis (no vs. yes)** | **0.01** | **0.16 (0.04, 0.67)** |  |
| Growth in mesencephalon (no vs. yes) | 0.83 | 1.15 (0.32, 4.19) |  |
| Growth in medulla (no vs. yes) | 0.34 | 1.70 (0.57, 5.06) |  |
| Growth in middle cerebellar peduncle (no vs. yes) | 0.99 | 1.01 (0.34, 3.00) |  |
| Tumor margin (ill-defined vs. well-defined) | 0.38 | 1.79 (0.49, 6.51) |  |
| Eccentricity within pons (no vs. yes) | 0.83 | 0.89 (0.30, 2.66) |  |
| Extrapontine extension (no vs. yes) | 0.97 | 1.03 (0.28, 3.74) |  |
| *Histologic/molecular* | | | |
| Biopsy/resection (no vs. yes) | 0.09 | 6.62 (0.74, 59.32) |  |
| Autopsy (no vs. yes) | 0.07 | 0.30 (0.08, 1.11) |  |
| **WHO grade (low vs. high)** | **0.004** | **0.15 (0.04, 0.55)** |  |
| **H3 K27M mutation (no vs. yes)** | **0.001** | **0.10 (0.03, 0.39)** |  |
| ***TP53* mutation (no vs. yes)** | **0.03** | **0.25 (0.08, 0.85)** |  |

| **Multivariable analysis** | | | |
| --- | --- | --- | --- |
| **Model** | **Covariates** | ***P value*** | ***Hazard ratio (95% CI)*** |
| 1 | **H3 K27M mutation (no vs. yes)** | **0.002** | **0.11 (0.03, 0.43)** |
|  | **Ring enhancement @ diagnosis (no vs. yes)** | **0.05** | **0.21 (0.04, 0.99)** |
| 2 | H3 K27M mutation (no vs. yes) | 0.06 | 0.19 (0.03, 1.09) |
|  | WHO grade (low vs. high) | 0.29 | 0.39 (0.07, 2.22) |
| 3 | **H3 K27M mutation (no vs. yes)** | **0.005** | **0.05 (0.01, 0.39)** |
|  | TP53 mutation (no vs. yes) | 0.24 | 0.45 (0.12, 1.70) |

*Abbreviation: WHO, World Health Organization.*
