# Supplementary material for: Clinical, imaging, and molecular analysis of pediatric pontine tumors lacking characteristic imaging features of DIPG
Source: Acta Neuropathol Commun. 2020 Apr 23;8:57. doi: 10.1186/s40478-020-00930-9 (PMC7181591; doi:10.1186/s40478-020-00930-9)
Supplement: Supplementary file 7 — Additional file 7: Table S5. Univariable analysis comparing clinical and radiographic variables between atypical DIPG without and with H3 K27M mutation. [file 40478_2020_930_MOESM7_ESM.docx]

**Supplementary Table 5.** Univariable analysis comparing clinical and radiographic variables between atypical DIPG without and with H3 K27M mutation.

| **Characteristic** | **Atypical DIPG without K27M mutation**  **(n = 21)** | **Atypical DIPG with K27M mutation**  **(n = 12)** | ***P*** |
| --- | --- | --- | --- |
| *Clinical* |  |  |  |
| Age @ diagnosis (yr), median (IQR) | 4.8 (2.5,7.6) | 9.0 (3.9,14.3) | 0.18 |
| Sex, no. (%) |  |  |  |
| Male | 12 (57%) | 6 (50%) | 0.73 |
| Female | 9 (43%) | 6 (50%) |  |
| Race, no. (%) |  |  |  |
| Black | 5 (24%) | 2 (17%) | 0.23 |
| White | 12 (57%) | 10 (83%) |  |
| Other | 4 (19%) | 0 (0%) |  |
| Symptom duration (mo), median (IQR) | 1.0 (0.5,6.0) | 2.0 (0.8,6.0) | 0.68 |
| Cranial nerve palsy, no. (%) |  |  |  |
| Yes | 17 (81%) | 11 (92%) | 0.63 |
| No | 4 (19%) | 1 (8%) |  |
| Pyramidal tract symptoms, no. (%) |  |  |  |
| Yes | 8 (38%) | 4 (33%) | 1.00 |
| No | 13 (62%) | 8 (67%) |  |
| Cerebellar symptoms, no. (%) |  |  |  |
| Yes | 13 (62%) | 7 (58%) | 1.00 |
| No | 8 (38%) | 5 (42%) |  |
| CSF diversion, no. (%) |  |  |  |
| Yes | 3 (14%) | 0 (0%) | 0.28 |
| No | 18 (86%) | 12 (100%) |  |
| Systemic therapy @ diagnosis, no. (%)  Yes  No | 11 (52%)  10 (48%) | 7 (58%)  5 (42%) | 1.00 |
| *Radiologic* |  |  |  |
| Tumor size (mL), median (IQR) | 20.5 (16.0,37.5) | 19.6 (16.7,28.6) | 0.67 |
| Ring enhancement @ diagnosis, no. (%) |  |  |  |
| Yes | 1 (5%) | 3 (25%) | 0.13 |
| No | 20 (95%) | 9 (75%) |  |
| Growth in mesencephalon, no. (%) |  |  |  |
| Yes | 6 (29%) | 3 (25%) | 1.00 |
| No | 15 (71%) | 9 (75%) |  |
| Growth in medulla, no. (%) |  |  |  |
| Yes | 13 (62%) | 6 (50%) | 0.72 |
| No | 8 (38%) | 6 (50%) |  |
| Growth in middle cerebellar peduncle, no. (%) |  |  |  |
| Yes | 10 (48%) | 4 (33%) | 0.49 |
| No | 11 (52%) | 8 (67%) |  |
| Tumor margin, no. (%) |  |  |  |
| Ill-defined | 14 (67%) | 10 (83%) | 0.43 |
| Well-defined | 7 (33%) | 2 (17%) |  |
| Eccentricity within pons, no. (%) |  |  |  |
| Yes | 9 (43%) | 7 (58%) | 0.48 |
| No | 12 (57%) | 5 (42%) |  |
| Extrapontine extension, no. (%) |  |  |  |
| Yes | 7 (33%) | 3 (25%) | 0.71 |
| No | 14 (67%) | 9 (75%) |  |
| *Abbreviations: IQR, interquartile range.* | | | |
