# Supplementary material for: Clinical, imaging, and molecular analysis of pediatric pontine tumors lacking characteristic imaging features of DIPG
Source: Acta Neuropathol Commun. 2020 Apr 23;8:57. doi: 10.1186/s40478-020-00930-9 (PMC7181591; doi:10.1186/s40478-020-00930-9)
Supplement: Supplementary file 8 — Additional file 8 Table S6. Molecular analyses and findings of the study cohort. [file 40478_2020_930_MOESM8_ESM.docx]

**Supplementary Table 6.** Molecular analyses and findings of the study cohort.

| **Case** | **Age** | **Gender** | **Histologic Diagnosis** | **Molecular Alterations** | **Testing Methods** |
| --- | --- | --- | --- | --- | --- |
| 1 | 0 | F | ETMR | C19MC amplification | Fluorescence *in situ* hybridization |
| 2 | 0 | M | Diffuse astrocytoma | H3/IDH-wildtype | Fluorescence *in situ* hybridization, immunohistochemistry, targeted sequencing |
| 3 | 1 | F | Angiocentric glioma | *MYB-QKI* fusion | Fluorescence *in situ* hybridization, immunohistochemistry, RT-PCR |
| 4 | 2 | F | Anaplastic astrocytoma | H3 K27M | Immunohistochemistry |
| 5 | 2 | M | Anaplastic astrocytoma | *HIST1H3B* K27M, *TP53* | Fluorescence *in situ* hybridization, immunohistochemistry, targeted sequencing |
| 6 | 2 | F | Angiocentric glioma | *MYB-QKI* fusion | Fluorescence *in situ* hybridization, immunohistochemistry, RT-PCR |
| 7 | 2 | M | Ganglioglioma | None identified | Fluorescence *in situ* hybridization, immunohistochemistry, targeted sequencing |
| 8 | 2 | M | Pilocytic astrocytoma | *KIAA1549-BRAF* fusion | Fluorescence *in situ* hybridization, RT-PCR, immunohistochemistry |
| 9 | 3 | F | Anaplastic astrocytoma | *HIST1H3B* K27M | Immunohistochemistry, targeted sequencing |
| 10 | 3 | M | Angiocentric glioma | *MYB-QKI* fusion | Fluorescence *in situ* hybridization, immunohistochemistry, RT-PCR |
| 11 | 3 | F | Embryonal tumor, NOS | None identified | Fluorescence *in situ* hybridization, immunohistochemistry, targeted sequencing |
| 12 | 4 | F | Angiocentric glioma | *MYB-QKI* fusion | Fluorescence *in situ* hybridization, immunohistochemistry, RT-PCR |
| 13 | 4 | M | Glioblastoma | *H3F3A* K27M, *TP53* | Immunohistochemistry, targeted sequencing |
| 14 | 4 | M | Embryonal tumor, NOS | None identified | Fluorescence *in situ* hybridization, immunohistochemistry, targeted sequencing |
| 15 | 4 | M | Low-grade glioma | None identified | Fluorescence *in situ* hybridization, immunohistochemistry, RT-PCR, targeted sequencing |
| 16 | 4 | F | Pilocytic astrocytoma | None identified | Fluorescence *in situ* hybridization, immunohistochemistry, RT-PCR, targeted sequencing |
| 17 | 4 | F | Pilocytic astrocytoma | *KIAA1549-BRAF* fusion | Fluorescence *in situ* hybridization, RT-PCR, immunohistochemistry |
| 18 | 5 | F | Angiocentric glioma | *MYB-QKI* fusion | Fluorescence *in situ* hybridization, immunohistochemistry, RT-PCR |
| 19 | 5 | M | Diffuse astrocytoma | *H3F3A* K27M, *TCF12* R423*/E610K | Fluorescence *in situ* hybridization, WES, RNA-seq, immunohistochemistry |
| 20 | 5 | M | ETMR | C19MC amplification | Fluorescence *in situ* hybridization |
| 21 | 7 | M | Diffuse astrocytoma | H3/IDH-wildtype | Fluorescence *in situ* hybridization, immunohistochemistry, targeted sequencing |
| 22 | 7 | M | Glioblastoma | *H3F3A* K27M, *ASXL2*, *PIK3R1* | Fluorescence *in situ* hybridization, WES, RNA-seq, immunohistochemistry |
| 23 | 8 | M | Diffuse astrocytoma | H3/IDH-wildtype | Fluorescence *in situ* hybridization, immunohistochemistry, targeted sequencing |
| 24 | 10 | M | Diffuse astrocytoma | *H3F3A* K27M | Immunohistochemistry, targeted sequencing |
| 25 | 12 | M | Anaplastic astrocytoma | *IDH1* R132C, *TP53* | Fluorescence *in situ* hybridization, immunohistochemistry, targeted sequencing |
| 26 | 13 | F | Anaplastic astrocytoma | *H3F3A* K27M | Immunohistochemistry, targeted sequencing |
| 27 | 13 | M | Glioblastoma | H3 K27M | Immunohistochemistry |
| 28 | 14 | F | Anaplastic astrocytoma | H3 K27M, *TP53* | Immunohistochemistry |
| 29 | 14 | F | Glioblastoma | *H3F3A* K27M, *TP53* | Immunohistochemistry, targeted sequencing |
| 30 | 14 | M | Pilocytic astrocytoma | *KIAA1549-BRAF* fusion | Fluorescence *in situ* hybridization, RT-PCR, immunohistochemistry |
| 31 | 16 | F | Glioblastoma | *H3F3A* K27M, *TP53* | Fluorescence *in situ* hybridization, immunohistochemistry, targeted sequencing |
| 32 | 16 | F | Glioblastoma | *IDH1* R132G, *TP53* | Immunohistochemistry, targeted sequencing |
| 33 | 17 | M | Anaplastic astrocytoma | *TCF12* I596T/E610K | Fluorescence *in situ* hybridization, WES, RNA-seq, immunohistochemistry |
